# Dual-functioning transcription factors in the developmental gene network of *Drosophila melanogaster*

**DOI:** 10.1186/1471-2105-11-366

**Published:** 2010-07-02

**Authors:** Denis C Bauer, Fabian A Buske, Timothy L Bailey

**Affiliations:** 1 Institute for Molecular Bioscience, The University of Queensland, Brisbane, Qld. 4072 Australia

## Abstract

**Background:**

Quantitative models for transcriptional regulation have shown great promise for advancing our understanding of the biological mechanisms underlying gene regulation. However, all of the models to date assume a transcription factor (TF) to have either activating or repressing function towards all the genes it is regulating.

**Results:**

In this paper we demonstrate, on the example of the developmental gene network in *D. melanogaster*, that the data-fit can be improved by up to 40% if the model is allowing certain TFs to have dual function, that is, acting as activator for some genes and as repressor for others. We demonstrate that the improvement is not due to additional flexibility in the model but rather derived from the data itself. We also found no evidence for the involvement of other known site-specific TFs in regulating this network. Finally, we propose SUMOylation as a candidate biological mechanism allowing TFs to switch their role when a small ubiquitin-like modifier (SUMO) is covalently attached to the TF. We strengthen this hypothesis by demonstrating that the TFs predicted to have dual function also contain the known SUMO consensus motif, while TFs predicted to have only one role lack this motif.

**Conclusions:**

We argue that a SUMOylation-dependent mechanism allowing TFs to have dual function represents a promising area for further research and might be another step towards uncovering the biological mechanisms underlying transcriptional regulation.

## Background

Site-specific transcription factors (TFs) that bind to the regulatory regions surrounding the target gene--so-called cis-regulatory-modules (CRMs)--are known to interact with the basal transcription complex to initiate transcription [1]. TFs can increase transcription by functioning as activators, or reduce transcription as repressors, respectively. The frequency and duration of the binding events is influenced by the concentration of the TF proteins, the binding affinities and location of the transcription factor binding sites (TFBSs) in the CRM, and the properties of the TFs themselves (e.g. effectiveness, competitive interaction with other TFs).

Modelling these binding events to quantitatively predict the resulting transcriptional output of the target gene has become increasingly successful [2-6]. The approaches model interaction of TFs and DNA using thermodynamic equations and predict the transcriptional response of the target gene as mediated by these interactions. A training algorithm is used to minimize the difference between the observed and predicted transcriptional response by adjusting the model parameters.

In previous research, thermodynamic models have been trained and tested on only one CRM [3,7]. Because the flexibility of the model is unlikely to be constrained sufficiently by the small amount of data from fitting only one CRM, it is possible that these models over-fit their input data, which would render them useless for the prediction of transcriptional output of other genes [6]. Indeed, in an earlier paper we showed that a large number of different model settings were able to produce nearly identical output, which supports the over-fitting hypothesis [8]. Gertz *et al. *[6] showed that the model predictions are more robust when models are trained on *multiple *synthetically generated CRMs. Hence, training on the regulatory sequence of multiple genes regulated by the same TFs should increase the quality of the generated model. Aiming at increasing the confidence in the trained thermodynamic model, Segal *et al. *[5] trained one model to fit the expression data of 44 developmental CRMs (20 genes) in *D. melanogaster*. Their model was able to fit about one third of the 44 CRMs with a correlation coefficient (CC) larger than 0.85 (where *CC *= 1 is perfect correlation and *CC *= 0 means no correlation). For the majority of the CRMs, however, the model fails to produce as good a fit, and the CC values are below 0.5 for a third of the data. They argue that the failure of their model can be attributed to missing higher-order interaction rules (like positive synergism) or missing input factors (especially activators).

An alternative explanation, however, is that not all 44 CRMs are regulated in the same way. Though all CRMs seem to be regulated by the same eight TFs, the role (repressor or activator) that the individual TFs have in the regulation of some CRMs might differ. Several experimental studies point to such "context-dependent" regulation for CRMs of developmental genes in *D. melanogaster *[9,10]. For example, Hunchback (Hb) is involved in regulating the even-skipped gene (*eve*), which is expressed in seven stripes along the body of the *D. melanogaster *embryo. It has been postulated that Hb takes the role of an activator in the CRM responsible for the expression of the second stripe, while being a repressor in the CRM regulating the third stripe [11].

To date, it is unclear what biological mechanism in *D. melanogaster *might explain how a TF switches from activator to repressor or *vice versa *(see Reinitz *et al. *[12] for a review). One hypothesis is that the TF itself does not switch its function, but that the altered outcome is an emergent property of the complex interactions of the system. For example, if a strong and a weak activator share a similar DNA binding profile, at high concentrations the weak activator can out-compete the strong activator and ultimately reduce transcription despite its role as activator [10]. However, this type of interaction should be fully captured by current thermodynamic models. Another hypothesis is the concentration-dependent switch, which proposes that a TF that is an activator at low concentrations can function as repressor at high concentrations (e.g. due to excessive aggregation) [13]. Yet another hypothesis is cooperativity, where the complex of two different TFs exert the opposite function as its individual components [10]. However, a protein-protein binding experiment of TFs and colocalization studies of the TFBSs failed to support this hypothesis [9,14,15].

In this article we suggest an alternative mechanism by which the developmental TFs in *D. melanogaster *can switch their regulatory roles. It has been shown that SUMOylation of individual TFs can lead to a loss or reversal of the regulatory function [16,17]. SUMOylation is a post-translational modification that attaches a small ubiquitin-like modifier (SUMO) covalently to a target protein [18].

The aims of this paper are twofold. Firstly, we investigate the validity of a SUMOylation-driven switching mechanism for the eight regulatory TFs in *D. melanogaster*. Secondly, we identify the role for each of the eight regulatory TFs that best describe the developmental gene network in *D. melanogaster*. To achieve this, we investigate the evidence for one or more TFs to have dual function, that is, function as activator for some CRMs and as repressor for others.

## Results and Discussion

### Analyzing the issues with state-of-the-art models

We first establish the ability of existing thermodynamic models of expression (Segal model [5] and Reinitz model [2]) to fit the *Drosophila *gap-gene expression data when only one role per TF is assumed. In order to measure the performance of a model, we record the correlation coefficient (CC) achieved when fitting all 44 developmental CRMs *simultaneously*. We assign Bicoid (Bcd), Caudal (Cad) and Torso-Response-Element (TorRE) as activator and Hunchback (Hb), Giant (Gt), Knirps (Kni), Krüppel (Kr) and Tailless (Tll) as repressor [5]. We herein refer to this particular role assignment as literature configuration. Simulated annealing (SA) is used to optimize the Reinitz model on the 44 CRMs and the accuracy is calculated from the resulting model. The accuracy of the Segal model on the same data is taken from predictions provided by Segal *et al. *[5].

Table 1 shows that neither the Segal model nor the Reinitz model are able fit all of the 44 CRMs *simultaneously*, with neither model achieving a CC of more than 0.6. The CC of the Segal model is more than twice as high as the one achieved by the Reinitz model. However, as indicated in the table, the Segal model has also far more free parameters. In particular, the Segal model learns the PWM parameters from the data. Since there has not been a detailed study examining the influence of the different model components on the ability to fit the data, one can only speculate to what degree the higher accuracy of the Segal model results from the large number of additional free parameters. It should be kept in mind that internal flexibility can lead to good fitting-accuracy despite wrong biological assumptions also known as "over-fitting" [8]. Note, as shown in the Additional file 1 Section 5 using these PWMs learned by the Segal model also improves the accuracy of the Reinitz model in fitting the 44 CRMs compared to the standard PWMs used in previous research [7]. Because we believe that the Reinitz model may be less prone to over-fitting when trained on small data sets, and because it is far faster to train, we use it in the subsequent experiments.

**Table 1:** Ability of the Segal model and Reinitz model to fit the Segal single-time data.

*model type*	*number of free parameters*	*mean simultaneously CC (SE)*
Segal	344	0.59 (0.009)
Reinitz	18	0.27 (0.008)

The **second **column shows the number of free parameters used in the models. The **third **column shows the average CC over all 44 developmental CRMs. The standard error is given in parentheses.

### Determining the regulatory role for each TF

Our next objective is to determine which of the 2^|TFs| ^= 256 possible configurations of how to assign the roles--activator or repressor--to each of the eight TFs fits the available data best. We consider an upper bound on accuracy by allowing the roles of all TFs to (potentially) be different for each CRM. A more reasonable upper bound would be given by allowing CRMs to choose from only a small number of configurations. Preferences for a certain role by a TF from each of these scenarios can suggest its true role for a given CRM. We also explore a sensitivity-based approach that measures the effect of changing the role of an individual TF.

There are 44 CRMs and 256 possible TF role configurations. We would like to consider all possible assignments of configurations to CRMs, and train models simultaneously using all CRMs. However, due to the huge number of possible assignments, this is technically infeasible. Instead, we train the Reinitz model using each CRM-configuration pair independently, and record the correlation between the observed CRM output and the model. This results in a 44 × 256 matrix of CC values, one for each CRM-configuration pair. We use this matrix in our three upper-bound and sensitivity analyses as described in the following paragraphs. We expect models trained this way to be heavily over-fitted and the CC to be overly optimistic. However, as described in Methods Sec. 0.4, by focusing on the strongest signals in the data, we are only extracting candidate role assignments for further testing in Sec. 0.1, where we train on all 44 CRMs again. We do not use the accuracy estimates otherwise.

**Figure 1:**
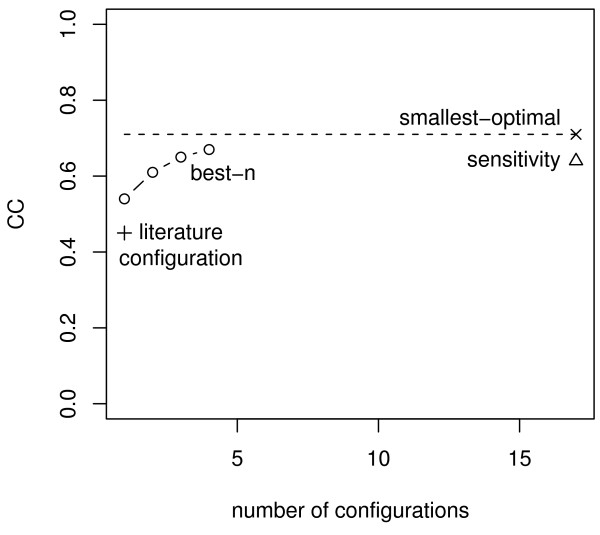
**Accuracy achieved using the configurations suggested by the role-determining methods**. The figure shows the average CC achieved when a Reinitz model was allowed to use as many different configurations as suggested by the different role-determining methods. BEST-N means the *n *∈ [1-4] configurations where chosen that have the best overall accuracy. SMALLEST-OPTIMAL means the model was allowed to use as many different configurations as necessary to fit each CRM optimally (here 17). Also shown is the average CC achieved over the 44 CRMs when the literature configuration was used. The standard error is ≤ 0.009 in all cases, hence no error bars are shown.

We first establish an upper bound on fitting accuracy by finding the best possible TF role configuration for each CRM *independently*. We do this using what we call the SMALLEST-OPTIMAL method, ∪, which identifies the TF role configuration(s) with best CC for each CRM *individually *and takes the union. The the smallest subset containing at least one optimal configuration for each CRM has size 17, with an average CC of 0.71 (standard error 0.006). Since the average CC is calculated over the set of optimal configurations with an *individually *trained model for each CRM, it represents the *upper bound *regarding the accuracy that can be achieved with freely altering the TF roles between CRMs (Figure 1, dashed line). We next look for a small set of configurations that most accurately fit the data. We are able to find optimum sets (in terms of total CC) with up to 4 configurations using an algorithm we call the best-n method, *β*. This method searches for the set of *n *configurations such that the total CC is minimum when each CRM is assigned one of the *n *configurations and trained independently from the other CRMs. As seen in Figure 1, the single best role assignment (*n *= 1) identified by the method already reaches 76% of the upper bound (average CC = 0.54, 0.007 standard error). By comparison, the literature configuration of TF roles achieves far lower accuracy (average CC = 0.45, *SE *= 0.009). These two configurations differ only in the role of Kr, which is a repressor in the literature configuration. Also shown in Figure 1, the accuracy converges to the upper bound with the number of additional configurations allowed. We use four configurations in the subsequent parts of the paper, which achieves an average CC of 0.67 (0.006), already 94% of the best CC achievable by the model on this data (upper bound).

Finally, the SENSITIVITY method, Δ, determines the role for each TF individually by identifying which change in role causes the largest change in accuracy between some pair of configurations. This is done independently for each CRM. This method results in 17 configurations (note, these 17 configurations are not identical with the 17 obtained by the smallest-optimal method). The average accuracy achieved by the optimal configurations is *CC *= 0.64 (*SE *= 0.006), slightly below the upper bound (Figure 1). For ten out of the 44 CRMs all three methods predict the same best configuration (see Additional file 1 Section 6. For the other 34 CRMs, the configuration predicted by at least one of the methods disagrees with those predicted by the other two. The agreement between the three methods is demonstrated for six CRMs in Table 2 (see Additional file 1 Section 6 for the full table).

**Table 2:** Assigning TF roles for different CRMs.

*CRM*	*Bcd*	*Cad*	*Hb*	*Tll*	*Gt*	*Kr*	*Kni*	*Torre*
Kr_CD1_ru	- (Δ)	+ (∪)	- (∪)	- (*β*)	- (*β*)	+	-	NA
eve_37ext_ru	-	+	+ (*β*)	+	+ (Δ)	NA	-	-
eve_stripe2	+	+ (Δ)	- (Δ)	-	-	-	-	NA
hb_anterior_actv	+	NA	+ (*β*)	-	- (Δ)	-	-	NA
kni_+1	+ (*β*)	- (Δ)	- (*β*)	-	+	- (Δ)	+	-
run_stripe5	+	+	-	-	-	-	-	- (*β*)

*roles*	s	+ (∪)	s (*β*)	- (∪)	s	s (*β*)	- (Δ)	s (*β*)

*confidence*	111	110	127	94	110	117	103	104

The **first rows **give the predictions for the TF roles for six of the 44 CRMs (see Additional file 1 Section 1 for the complete set of predictions). The roles for each TF (columns) are determined by majority vote of the three methods: "∪" - SMALLEST-OPTIMAL, "*β*" - BEST-N and "Δ" - SENSITIVITY. "+" means activator, "-" repressor, respectively and "NA" indicates that no "strong" role prediction could be made for the CRM. The disagreeing method, if any, is shown in brackets. The **second last row **gives the overall prediction of the role of the TF: activator, repressor or switcher, "s" (see main text for method). The **last row **shows the number of "strong" role predictions summed over the 44 CRMs and three methods (total of 132).

The disagreement between the methods calls for the construction of a voting ensemble. We aim to integrate the different properties of best accuracy (∪), smallest number of different configurations (*β*) and strongest signal (Δ) in an ensemble by combining the three methods using majority vote. Table 2 shows the role assignment over all CRMs as determined by this ensemble (Table 2, last row). To define a single role for each TF we first combine for each method individually the evidence for all CRMs and then build the majority vote from them. We combine the evidence by defining a TF to be an activator or repressor if for more than  of the CRMs the TF was favoured as activator and repressor, respectively (see Methods Sec. 0.4.2). If for fewer than  of the CRMs the function agreed, we define the TF to switch roles. Based on this approach we propose that Cad is consistently an activator, Tll and Kni are consistently repressors, and Bcd, Hb, Gt, Kr and Torre function as switching or "dual-functioning" TFs. As shown in Table 2, Hb and Kr are the TFs with the "strongest" role predictions (see Methods Sec. 0.4) and provide hence the strongest evidence for dual function. We use both TFs as candidates to address the question how the dual function impacts on the model accuracy.

### 0.1 Improving the data fit using dual roles

The above results based on fitting the Reinitz model to individual CRMs strongly suggest that TFs Hb and Kr may play different roles for different CRMs. In this section, we further explore this possibility by training models *simultaneously *on all 44 CRMs. We train three models, which we call HbDual, KrDual and HbKrDual, that allow one or both of Hb and Kr to be assigned a specific role (activator or repressor) for each individual CRM. For each CRM, the role of Hb, Kr or both is determined by their roles predicted by the SENSITIVITY method (see Additional file 1 Section 3 for the list of CRMs to which Hb or Kr is attributed as activator). The roles of the other TFs are fixed at the literature configuration. As before, we use the CC of the fit of the model to the data as a measure of accuracy.

Table 3 shows that allowing for dual function improves the ability of the Reinitz model to fit the data substantially. Allowing Kr to switch its role boosts the accuracy by 30% to *CC *= 0.35 compared to the Reinitz model using the literature configuration (*CC *= 0.27). An even better fit is obtained when Hb adopts dual function (*CC *= 0.37). Allowing both Hb and Kr to switch roles further increases the data fit, however to a lesser degree, resulting in a best accuracy of *CC *= 0.38 (40% improvement).

**Table 3:** Improvement in the ability to fit the data when dual function for Hb and Kr are allowed.

*model type*	*number of configurations*	*number of free parameters*	*mean simultaneously CC (SE)*
Reinitz	1	18	0.27 (0.008)
Reinitz KrDual	2	19	0.35 (0.009)
Reinitz HbDual	2	19	0.37 (0.007)
Reinitz HbKrDual	4	20	0.38 (0.007)

Segal	1	344	0.59 (0.009)

Each row shows the CC for the Reinitz model using the literature configuration, and additionally Hb, Kr or Hb and Kr as switching TFs, respectively. The results are contrasted to the Segal model given in the last row. The **second **column shows the number of different configurations in the approach. The **third **column indicates the number of free parameters in the model. The **fourth **column shows the average CC when training on all 44 CRMs *simultaneously*. The reported results for the Reinitz model are averaged over five independent runs.

For each TF that is allowed to switch, one additional free parameter is added to the model, since the model must contain an "effectiveness" parameter, , for each role of switching TFs. The complexity of the model is increased further by the fact that it is essentially clustering the CRMs into two or four classes (according to the roles of Hb and/or Kr), and modeling each class separately. However, each of these class models shares all but at most two of its free parameters with the other class models.

The relatively small gain from HbDual to HbKrDual has two implications. *Firstly*, there must be a large set of CRMs that benefit from either switch. This is most likely due to TFs now developing their full effectiveness rather than compromise to limit the negative effects from wrongly assigned roles. Hence, the effect of assigning the "correct" role to a particular CRM can also prove beneficial for other CRMs. The *second *implication is that despite the global improvement, there are TF-specific gains observable, which can only be achieved if both TFs can serve their CRMs correctly. The accuracy of each individual CRM are provided in Additional file 1 Section 1.

Additional file 1 Section 3 shows the expression profile of the CRMs attributed to Hb (or Kr) as activator, demonstrating that the assignment is not "trivial", that is CRMs with activation coinciding with the protein expression of Hb are not all assigned to the Hb activator set. For example, *kni_+1 *and *kr_CD2_ru *have a transcriptional output at the AP position where Hb is expressed, yet they do not require Hb to be an activator.

**Figure 2:**
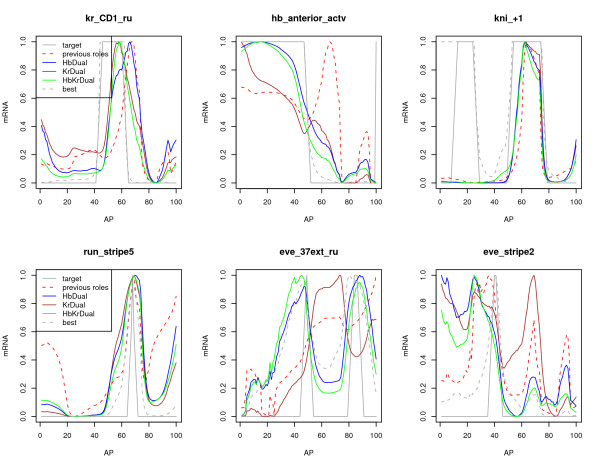
**Performance improvement of models using dual functioning TFs**. Each panel shows for six representative CRMs the observed output of the CRM (solid grey) compared to the normalized predicted shape using different TF roles in the Reinitz model. The **grey dashed **line shows the prediction for the best role assignment and individual training on the CRM. In **dashed red **is shown the performance when using the literature configuration and trained simultaneously on all 44 CRMs. The **solid coloured **lines show the prediction of HbDual KrDual and HbKrDual, respectively, trained *simultaneously*. The model with the best over-all performance is displayed from the five independent repeats.

These three effects are visualized in Figure 2. A model with literature configuration assignment achieves a CC of 0.13 for *kr_CD1_ru*, which can be improved by HbDual to 0.41, however the peak of the prediction is clearly shifted posterior by both models. KrDual corrects this shift considerably increasing the accuracy to 0.676, however has an extension of the activation anterior, which can only be corrected by the HbKrDual model. The latter allows for a stronger repressing Hb at the required position (40% AP), achieving a CC of 0.681. Similarly, the initial accuracy of *hb_anterior_actv *of 0.36 can be improved by KrDual reducing the falsely predicted posterior peaks but only HbDual and HbKrDual can improve the accuracy to capture the shape with *CC *= 0.93 correctly. The CRM *kni_+1 *is an example for which accuracy improvement from HbDual, KrDual and HbKrDual are identical. For these three models the role of either Hb or Kr or both deviated from the literature configuration. There are also accuracy gains due to indirect effects such as a more accurate parameter estimation, as shown with *run_stripe5*, for which the roles of Hb and Kr do not change with regard to their respective literature configuration. *eve_37ext_ru *is an example where only the switch of one TF increases accuracy, while (additional) switching of the other TF results in negative effects. The Reinitz model initially achieves a CC of 0.22 on *eve_37ext_ru*, which improves to 0.58 by HbDual but then decreases to 0.48 in HbKrDual. This might be due to a wrong role assignment obtained by the SENSITIVITY method for Kr. The same reason may be responsible for a decrease in accuracy on a specific CRM for any investigated Dual model as exemplified with *eve_stripe2*. The question remains if other TFs than the investigated eight are involved in the regulation of developmental genes and can account for the observed behavior of Hb and Kr.

### Determining the involvement of other TFs in the regulation

In this section we test if other regulatory TFs might be involved in the regulation of developmental genes. Since these (additional) TFs are not included during the training of the model, the proposed dual-functioning TFs might mimic or compensate for their task rather than being indeed dual-functioning *in vivo*.

If such a site-specific TF is missing from the set of regulators, it should have TFBSs in the regulatory regions of *D. melanogaster*. Searching in the sequence of all 44 developmental CRMs for enriched sites might indeed retrieve a yet unknown TF that is involved in the regulation. However, without the TF's expression profile we would not be able to make any inferences whether the TF's involvement is a better explanation for the observed regulatory differences than the dual-functioning of one of the eight regulatory TFs. Finding a motif that is enriched in one set of CRMs relative to the other set, however, would be a potential explanation for why the CRMs act differently.

In the previous section, we already identified two sets of CRMs with different regulatory mechanisms, one with Hb favoured as activator and the other where Hb is preferred a repressor. If a missing TF, rather than the dual role of known TFs, is responsible for the difference in regulation between the two CRM sets, the TFBSs of this unknown TF should be enriched in one set, while underrepresented or background distributed in the other. The enrichment should be more pronounced than when comparing to non-regulatory sequence. The same should be the case with the set of CRMs favouring divergent roles for Kr. We use CLOVER [19] to identify which *Drosophila *TFs with known binding profiles are statistically overrepresented or underrepresented in the sequences of the Hb_act set, compared to the Hb_rep set (see Methods Sec. 0.6 for more details), and we do likewise for Kr.

The enrichment *p*-values for the Hb and Kr sets, compared to all regulatory genomic regions in *D. melanogaster*, are shown in Table 4 We identify 12 out of the 76 known site-specific TFs to be over-represented in one of the four sets of CRMs. Only three are differentially enriched--that is overrepresented in the activator set and underrepresented or background distributed in the repressor set (or *vice versa*).

**Table 4:** TFs with enriched TFBSs in CRMs where Hb or Kr are activators or repressors.

*TF*	*Hb*		*Kr*	
	*p-value for set*		*p-value for set*	
	***act***	***rep***	***act***	***rep***

# of CRMs	17	27	11	33
Abd-B	0.0	0.0	0.0	0.0
Deaf1			0.079	0.005
His2B	0.048	0.0	0.0	0.058
Hsf*	0.085	0.008	**0.0**	**0.231**
Kr	0.0	0.0	0.0	0.0
Bcd	0.0	0.0	0.0	0.0
Br-Z4^+^			**0.821**	**0.006**
Cad	0.002	0.0	0.0	0.0
Hb	0.032	0.0	0.0	0.0
Kni	0.032	0.01	**0.702**	**0.0**
Tll	0.003	0.0	0.0	0.0
Ttk	0.02	0.0	0.003	0.0

The first row shows the number of CRMs in each set. Each following row shows a TF out of the 76 tested for which a Clover [19] analysis resulted in a significant over- or under-representation in the sequences where Hb is preferred as activator (**second **column) or repressor (**third **column) or Kr preferred as activator (**fourth **column) or repressor (**fifth **column), respectively. Highlighted in bold are the cases with differential enrichment between the activator and repressor set. The TF marked with "*" is not expressed during the developmental time points *C13 *and *C14 *as determined from *in-situ *staining images http://www.flyexpress.net. The TF marked with "^+^" has a binding profile that is very similar to Bcd. Empty cells indicate that the TF was not significant for the sequence sets of Hb.

For the Hsf and Kni motifs, one set of sequences was enriched for the motif with a CLOVER[19]*p*-value (uncorrected) of 0, which actually represents a *p*-value of "no greater than 0.001" [19]. Since 76 motifs were considered, the corresponding Bonferroni corrected *p*-value is 0.076, so we can state that these motifs are enriched at that significance level. For these motifs, the other sets of sequences had uncorrected *p*-values greater than 0.5, and corrected *p*-values close to 1, so these motifs were definitely not enriched in the alternate sequence sets. In the case of the Br-Z4 motif, one set of sequences has an uncorrected *p*-value of 0.006, which is not significant when corrected. Nonetheless, we suspect that this motif is enriched in that set of sequences, and not enriched in the other set, where the uncorrected *p*-value is 0.821.

However, based on prior knowledge of the functions of these three candidates, none of them seems to be a missing TF for the regulation during development. Hsf does not seem to be present during gastrulation, according to staining images from http://www.flyexpress.net, and hence cannot influence the expression of the developmental genes. Br-Z4 has a binding profile similar to Bcd (TOMTOM[20] p-value = 0.0091, see Additional file 1 Section 2), which is enriched in both sets. The under-representation of Br-Z4 is hence likely due to the higher proportion of the Bcd-version of the motif rather than the true absence of any binding sites of Br-Z4. Finally, the under-representation of Kni in the Kr_act set can be explained by the observation that a large proportion of the CRMs that prefer Kr to be an activator produce expression that coincides with the AP position of highest Kni concentration (see Additional file 1 Section 3). Since Kni is consistently a repressor, its binding would inhibit activation, which makes the absence of Kni TFBSs in those CRMs favourable and explains the under-representation of Kni sites in the Kr_act set. It is noteworthy that binding sites for Slp1, which was suggested by Andrioli *et al. *[21] to be involved in the regulation of some of the CRMs studied here, are neither over- nor under-represented) in any of the CRMs (data not shown). Hence, regulation by Slp1 appears not able to account for the (apparent) differing regulatory function of the other TFs.

### Analyzing SUMOylation as the mechanism for dual-functioning TFs

As described in the previous section, we failed to find an enriched TF binding motif, which strengthens the hypothesis of dual-functioning TFs. In this section we explore the protein sequences of the TFs proposed to be dual-functioning to see if there exists a biological marker that discriminates them from proteins proposed to have a single role. We therefore search for short motifs (≈5 *aa*) common in all protein sequences of TFs with dual function using MEME [22] for finding one occurrence per sequence, "OOPS". In order to identify motifs that generally appear in the eight regulatory sequences, we also scan the protein sequences of TFs predicted to have single roles.

**Figure 3:**
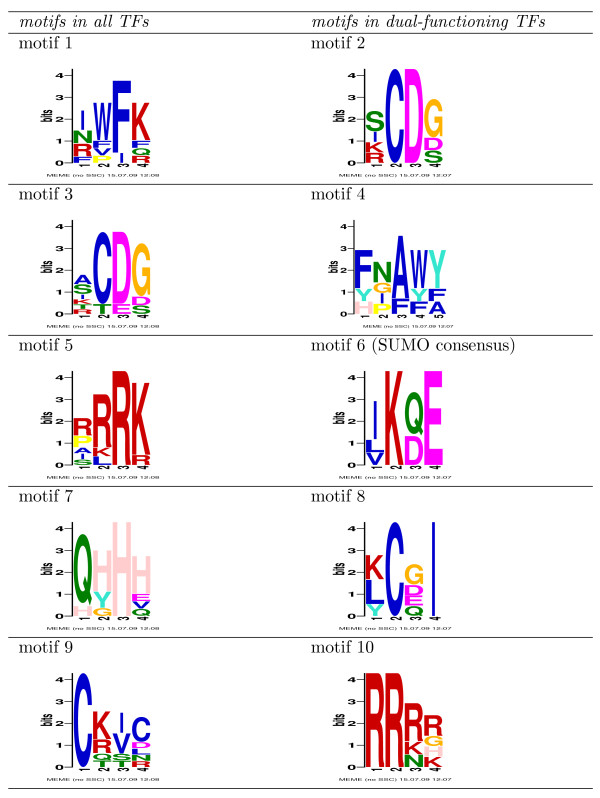
**Motifs found in protein sequences according to MEME**. Each panel shows the logo representation of a motif found in the protein sequence of all eight regulatory TFs (left column) or all TFs predicted to have dual function (right column). Motifs were found using MEME [22] in "OOPS" mode with a minimum sequence length of four and a maximum of six.

The results of these scans are shown in Figure 3. As motifs 2 and 10 appear also in the protein sequence of TFs predicted to have a single role (motifs 1 and 5), they can hence be disregarded as marker. Motif 4 does not have a high information content because no sequence position was fully conserved amongst the five protein sequences, which indicates that the motif may only represent noise. This narrows the search for a marker down to two motifs: Motif 6, which is Ψ *K*. *E*, where "Ψ" represents a aliphatic amino acid (I, L, V) and "." represents any amino acid, and motif 8, which is Φ*C*. *I*, where "Φ" represents a hydrophobic amino acid (K, L, Y). While a literature search revealed no information about motif 8, motif 6 was in fact identified as the known SUMOylation consensus motif. SUMOylation is the post-translational modification, where a SUMO protein, is covalently attached to the target protein. As a matter of fact, SUMOylation has been described to alter the transcriptional function of a TF. Additional file 1 Section 4 shows the positions of the SUMOylation consensus motifs with respect to other protein domains in the eight TFs [23].

Table 5 summarizes for each TF the roles as suggested by this research and the presence of SUMOylation consensus motifs. All of the TFs predicted to switch roles contain a SUMOylation consensus motif and all TFs predicted to have a single role lack this motif. The suggested role for Cad, Hb, Tll, Gt and Kr is in agreement with the literature, which used distinct methods to derive this information.

**Table 5:** Role of TFs in comparison with presence of SUMOylation consensus motif and the role reported in the literature.

	*Bcd*	*Cad*	*Hb*	*Tll*	*Gt*	*Kr*	*Kni*	*TorRE*
**roles**	**s**	**+**	**s**	**-**	**s**	**s**	**-**	**s**

**Number of SUMO sites**	**1**	**0**	**2**	**0**	**1**	**2**	**0**	**1**

Perkins *et al. *[29]	+	+	s	-	s	s	-	NA
Schroeder *et al. *[9]	+	+	s	-	-	(s)	-	+
Rivera-Pomar *et al. *[35]	+	+	s	-	-	s	-	NA
Sanchez *et al. *[36]	+	+	s	-	-	-	-	NA
Jaeger *et al. *[37]	+	+	s	-	s	s	s	NA

The **first row **indicates the role we assign to the TFs, where "+" indicate activators, "-" indicate repressors, and "s" indicates a TF switching roles. The **second row **shows the number of sites found in the protein sequence of the TF that match the SUMO-consensus motif. The **last rows **summarizes the roles for the TFs previously reported in the literature.

It is believed that the functional role of a TF can be mediated by the SUMO-dependent interaction with different cofactors. Valin *et al. *[24] validated this hypothesis for Sp3, a Zinc finger C2H2-protein like Hb, where SUMOylation promotes the interactions with a corepressor protein causing the complex to repress transcription, whereas a non-SUMOylated Sp3 protein promotes transcription. SUMOylation has also been shown to increase transcriptional activation [25,26]. Another related example is Ikaros, the human homologue of Krüppel (Kr), whose ability to repress is reduced when SUMOylated [27]. The loss or reduction of repressor function in concert with the competition for binding sites with a stronger repressor can result in overall activation [10]. Furthermore, Stielow *et al. *[28] showed that a SUMO-modified TF can silence genes by triggering the formation of local heterochromatin-like structures. Specifically, they showed that a modified Sp3 protein recruits chromatin remodelling proteins. It remains to be shown whether one of these mechanisms can account for the postulated functional change in the role of the TFs and whether an interaction with SUMO is both protein-concentration and CRM dependent.

## Conclusions

In this study we investigated the developmental gene network of *D. melanogaster*. Using a thermodynamic model, we studied the effect of TFs taking opposite roles for distinct sets of CRMs. We identified five TFs with potentially dual roles from the data and investigated the two TFs that provide the strongest evidence for having dual function, Hunchback (Hb) and Krüppel (Kr). Our identification of these two TFs as potentially acting both as activators and repressors agrees with previously reported evidence [9,29]. We show that the accuracy with which our chosen thermodynamic model can be fit to existing gene expression data increases by 40% when both Kr and Hb are allowed to have dual function, and by 30% and 37% respectively when Kr or Hb are allowed to switch.

Our results do not support the previously hypothesized role of Hb as a repressor in the CRM regulating the third and seventh stripe of *eve *expression, *MSE3+7 *[11]. We predict that Hb is an activator for *MSE2 *and *MSE3+7 *and a repressor for the remaining *eve *stripes. Interestingly, *MSE2 *and *MSE3+7 *are located upstream of the *eve *gene, while the other MSEs are located downstream. Our findings also disagree with the concentration-dependent switching mechanism proposed by Papatsenko *et al. *[13], as CRMs requiring Hb to act as repressor do not drive peaks in expression at locations in the embryo with the highest concentration of Hb protein.

We also explore *in silico *the possibility that another *known *site-specific TF might be binding to the CRMs used in our study. No known TF DNA-binding motif is differentially enriched in the CRMs in which Hb (or Kr) appears to act as an activator compared with those where it appears to act as a repressor (or *vice versa*). This of course does not rule out the possible involvement of some other TF whose DNA-binding motif is not yet known; Nor does it address the possibility of an unknown non-DNA-binding TF being the mechanism required to explain the inability of the thermodynamic model to successfully fit the data. We hypothesize a SUMOylation-propelled mechanism for the TF role-switch in the different CRMs. Support for this idea is provided by the fact that SUMO consensus motifs are present in exactly the subset of TFs that our thermodynamic model-based study predict to be role-switchers. Our hypothesis is in line with prior evidence linking SUMOylation to the modulation of transcriptional activity of TFs [30-32]. This previous work has shown that the SUMOylation status of a TF affects which cofactors it can interact with, thus modulating its affect on transcription. Our hypothesis is also made more plausible by the fact that the SUMO homologue in *Drosophila*, Smt3, is uniformly distributed throughout the embryo [17,33]. In conclusion, our results suggest that current assumptions of a uniform, fixed regulatory mechanism for all developmental CRMs should be revised towards a model allowing for dual roles to improve prediction accuracy. Furthermore, our proposed mechanism of a SUMOylation-dependent switch represents a promising area for further research, and might be another step towards uncovering the biological mechanisms underlying the transcriptional regulation of the *D. melanogaster *developmental network. A full knowledge of this system will be one of the breakthroughs needed for a quantitative understanding of transcription and ultimately the development of accurate predictive models of gene expression from DNA sequences.

## Methods

### 0.2 Static, thermodynamic models of transcription

We consider two thermodynamic models referred to as the Reinitz model [3] and the Segal model [5]. In a nutshell, both approaches model the transcription rate of a gene as a function of a vector of free model parameters, Θ, and a vector of model inputs, **X**. The model inputs comprise the set of regulatory TFs, the protein concentrations of these TFs and a set of log-odds (base two) scores describing the TF binding affinity along the controlling CRM. Latter is obtained by scanning the sequence of the CRM using a position weight matrix (PWM) representation of the TF binding preference and a PWM scanner, e.g. FIMO [22]. In addition, the Reinitz models requires a pre-specified role - activator or repressor - for each of the TFs as input, while the Segal model optimizes the role during training. We represent the role of the *n *TFs by a binary vector *ϕ *= ⟨*r*_1_, *r*_2_, ⋯, *r*_n_⟩, where *r_i _*= 1 indicates TF *i *is an activator, and *r_i _*= -1 a repressor, respectively. We refer to *ϕ *as a "configuration".

While the Segal model uses the log-odds binding scores at all positions in the CRM, the Reinitz model discretizes them by identifying individual (high-scoring) TFBSs when applying a PWM score threshold, *t*. We call a set of positions and log-odds scores "TFBS-map".

The set of free parameters, Θ, of the Reinitz model is considerably smaller than that of the Segal model. Reinitz's set of parameters comprises a maximum association constant (*K*), an "effectiveness" constant (*E*) for each TF, the Gibbs free energy threshold (*G*_0_) of transcription and the maximal transcription rate (*R*_0_). This comes to 2*n *+ 2 parameters in Θ_Reinitz_, where *n *is the number of TFs. In contrast, |Θ_Segal_| = 3*n *+ 3|*W*| + |*C*|, where |*W*| is the overall sum of the length of each PWM for the TFs and |*C*| is the number of CRMs. The three parameters per TF comprise the concentration scaling factor, which is comparable to *K*, and the expression contribution, which is comparable to *E*, while the third free parameter is the self-cooperativity value, which has no counterpart in the Reinitz model as synergy between TFs is not modeled there. The second term of the sum covers free parameters for the PWM, which are optimized for each TF during training. Finally, for every CRM, *c*, in the data set there is a free parameter describing its basal transcription rate, .

### Data sets

We use the *D. melanogaster *gap-gene data set [5]. The data set comprise spatial and temporal concentration patterns of eight TF proteins: Bicoid (Bcd), Caudal (Cad), Giant (Gt), Hunchback (Hb), Knirps (Kni), Krüppel (Kr) Tailless (Tll) and Torso-Response-Element (TorRE). The data set also contains estimates of the transcriptional response mediated by 44 CRMs, as measured by mRNA levels generated by lacZ-reporter constructs containing each of the CRMs. The protein and mRNA measurements are made at the same developmental time points at multiple points across entire *D. melanogaster *embryos. The data set also contains the DNA sequence for each of the 44 CRMs. We use a PWM describing the binding preferences of the TF and the motif scanning program FIMO [22] to predict discrete TFBSs within the regulatory regions. As described in Sec. 0.2, an important feature of the Segal model is to adjust the known PWMs. Altering the binding preference of each TFs allows the model to better describe the observed data but may, at the same time, introduce a bias towards the data set at hand. We use both the standard ("off-the-shelf") PWMs [7], and the "tweaked" PWMs to generate a TFBS-map. Since Segal *et al. *[5] used a uniform background frequency to calculate the log-odds score we also use a uniform background for the tweaked PWMs, and a *D. melanogaster *specific background frequency, *F_A _*= *F_T _*= 0.297 *FC *= *FG *= 0.203, when using the "off-the-shelf" PWMs. The Reinitz model normalizes the PWM scores for the TFBSs of TF *a *in the data set by the maximal possible score, , which is the sum of the largest log-odds score in every column of the PWM. We use a PWM threshold *t *= 9 *bits*.

The Reinitz model allows for distance-dependent repression, where the effect of activators are reduced when they are within *d *base pairs of an occupied repressor site. The Segal model does not have a distance-dependent repression mechanism. Instead, a repressor reduces the effect of all bound activators within the CRM. If the distance parameter in the Reinitz model is set to a number larger than the extent of the CRM, the two models should exhibit the same behavior with respect to the repressor function.

### 0.3 Training on multiple CRMs and different configurations

To measure how well each configuration can fit each CRM in the Segal single-time data, we train one Reinitz model for each possible configuration and each of the 44 developmental CRMs *individually *using the SA optimizer with geometric cooling schedule [23]. SA was allowed to optimize for 1000 iterations and the resulting CC is averaged over five independent repeats. This generates a 44 × 256 matrix with the average CC values of each configuration on each CRM.

By training a Reinitz model on each CRM *individually*, rather than training one model on all CRMs *simultaneously *we obtain an upper bound of how well the Reinitz model can fit each CRM. The small size of the training data set almost certainly causes the model to over-fit and, hence, the resulting CC values are likely overly optimistic. So, failure to produce good CC values is hence an even stronger indication for that the model is not able to fit the CRM with a given configuration of TF roles. To determine the roles for a TF from the CC matrix, we use the vote of three different methodologies, each focusing on a different aspect of the data, therefore avoiding the bias and isolating the signal picked up by the majority. These methodologies are called "Role-determining methods" and are described in the next section.

### 0.4 Role-determining methods

We employ three different role-determining approaches: the SMALLEST-OPTIMAL method, ∪; the Best-N method, *β*; and the SENSITIVITY method, Δ. Each of these approaches is based on analyzing how well the Reinitz model fits the data for an individual CRM and configuration.

#### Smallest-optimal (set of configurations)

The smallest optimal set of configurations is chosen such that each individual CRM is assigned to its optimal configuration, determined by the best *individual *training accuracy (CC). If a particular TF has no influence on the regulation of a CRM, that is if all the configurations-pairs with the TF in different roles have a similar CC--that is, if the difference in CC between the two methods, Δ, is less than a constant, ϵ--the role of the TF is set to "NA". We use ϵ = 0.1. Any role predictions other than "NA" are defined as "strong" in this method. While this method is guaranteed to identify the configurations with the absolute best accuracy, we expect the achieved CC values to be over-fitted, hence identifying the best performing CC might be noisy.

#### Best-N

The BEST-N method chooses the set of *n *different configurations that jointly achieve the optimal performance on all CRMs. This greedy approach is computationally expensive since there are  combinations to test and hence, in this form, it is only feasible up to *n *= 4. However, since we assume that a small set of different configurations is able to explain the regulation of the 44 CRMs, there may be no need for an exhaustive calculation for *n *> 4. This approach always assigns a CRM to a configuration, we therefore receive "strong" role predictions for each TF in every CRMs. Similar to the SMALLEST-OPTIMAL method, the reliance on the accuracy measure might be a problem given that we assume them to be over-fitted.

#### 0.4.1 Sensitivity

The SENSITIVITY method identifies a particular role for each TF in a CRM rather than the best (set of) configuration(s). The role *r *is chosen for TF *t *in CRM *c *if it is "critical"--that is, if switching to it causes the largest increase in fitting accuracy. For a given CRM-TF pair, we compute the difference in performance between all pairs of configurations that differ only in the role of the TF, and assign the TF the role that causes the maximum increase in fitting accuracy. This is described in detail in the next paragraph. Let *ϕ *be a configuration specifying the roles of each of the 8 TFs. Let *A*(*ϕ*, *t*) be *ϕ *with the role of TF *t *set to "activator", whereas *R*(*ϕ*, *t*) sets the role of *t *to "repressor". Let *CC*(*c*, *ϕ*) be the correlation coefficient of the expression model fit to CRM *c *using configuration *ϕ*. Then, for CRM *c*, the maximum improvement in fit accuracy we can obtain when we switch *t *from "repressor" to "activator" is given by

We assign the role of *t *according to the sign of the maximum difference in accuracy, making no assignment if the difference is too small:

We define a role prediction as "strong" in this method if the absolute value of ΔCC *>*0.1.

#### 0.4.2 Defining single- and dual-functioning TFs

To identify if a TF has a single role for each CRM, we first combine for each method individually the evidence for all CRMs by counting how many times this particular TF was preferred as activator and repressor and calculating a fold-change over one or the other role. We translate this fold-change to mean the TF is a consistently an activator or repressor if more than  of the CRMs favoured the TF as activator and repressor, respectively. If for less than  of the CRMs the function agreed, We define the TF to switch roles. We then combine the evidence for single- or dual-function obtained for the role-determining methods individually by building a majority vote over the assigned functions.

### 0.5 Training the model when dual-function is allowed

To perform the training with dual-functioning TFs, we first use the SENSITIVITY method (described in Sec. 0.4.1) to determine the set of CRMs for which the TF was favoured in one or the other role by training a model on every CRM-configuration pair. CRMs where no role was determined by the SENSITIVITY method were assigned to configurations using the TF in its literature configuration. The TFBS-map is then adjusted such that only the appropriate version of the TF--activator or repressor--can bind to the TFBSs in the map according to what the SENSITIVITY method had determined for this CRM. Note, the protein concentrations remain the same for the TF irrespective of its function. We then train on all 44 CRMs *simultaneously *as described in Sec. 0.3, except that now two free parameters describe the effectiveness of the TF as activator, *E*_activator_, and repressor, *E*_repressor_, respectively.

### 0.6 Enrichment analysis

We perform motif enrichment analysis in order to discover if other site-specific TFs might be involved in the regulation of the developmental genes in *D. melanogaster*. The assumption is that if a TF is either directly involved in the regulation of the genes, or mediating role-switching by one of the eight TFs, it will have more binding sites in the set of CRM sequences that require the TF to be an activator compared to the set of sequences that require it to be a repressor (or *vice versa*). We call TFs that are over-represented in one set of CRM sequences compared to the other "differentially" enriched.

We use CLOVER [19] to calculate the *p*-value for over- or under-representation of TFBSs given a specific TF and a set of sequences compared with all intergenic regions in *D. melanogaster*. We collect two sequence sets, which were predicted to be subject to a different regulatory mechanism: set *A *were Hb was predicted to be an activator and set *R *where Hb was predicted o be a repressor, for Kr respectively. For each of these sequence sets we run CLOVER [19] to identify TFs whose TFBSs are enriched in one or the other set. We call a TF to be over-represented if the *p*-value is below 0.01 and under-represented if the *p*-value is above 0.99. We report all TFs with known binding specificity taken from http://www.danielpollard.com/matrices.html and Slp1 from JASPAR [34] (76 in total) that are overrepresented in set *A *or *R*. For those TFs to be called "differentially" enriched we require the difference in *p*-values between set *A *and *R *to be larger than 0.2.

## Authors' contributions

DCB researched and carried out the experimental work under the supervision of TLB. FAB designed the BEST-N method. The initial manuscript draft was written by DCB, and refined by FAB and TLB. All authors read and approved the final manuscript.

## Supplementary Material

Additional File 1 **Supporting material for the article**. The file contains Sections 1 to 6 referred to in the article. Click here for file
