# Supplementary material for: Dual-functioning transcription factors in the developmental gene network of Drosophila melanogaster
Source: BMC Bioinformatics. 2010 Jul 2;11:366. doi: 10.1186/1471-2105-11-366 (PMC2912886; doi:10.1186/1471-2105-11-366)
Supplement: Additional File 1 — Supporting material for the article. The file contains Sections 1 to 6 referred to in the article. [file 1471-2105-11-366-S1.PDF]

Section 1 : Performance improvement for dual models

CRM	*previous CC*	*HbDual CC*	*KrDual CC*	*HbKrDual CC*	Δ *Hb*	Δ *Kr*	Δ *HbKr*	*role Hb*	*role Kr*
knrl_+8	-0.507	0.231	0.203	0.161	0.739	0.711	0.669	0	0
run_stripe3	-0.223	-0.020	0.129	0.419	0.203	0.352	0.642	0	1
gt_-1	-0.480	0.088	-0.492	0.121	0.568	-0.012	0.600	1	0
hb_anterior_actv	0.358	0.926	0.822	0.928	0.568	0.464	0.571	1	0
Kr_CD1_ru	0.125	0.413	0.676	0.681	0.288	0.551	0.557	0	1
hb_centr__post	-0.150	0.130	-0.286	0.400	0.280	-0.137	0.549	1	1
Kr_CD2_ru	0.058	0.311	0.514	0.535	0.253	0.456	0.477	0	1
slp2_-3	0.388	0.738	0.751	0.772	0.351	0.363	0.385	1	0
eve_37ext_ru	0.218	0.582	0.020	0.479	0.364	-0.198	0.261	1	0
cad_+14	-0.100	0.174	0.028	0.105	0.273	0.128	0.205	1	0
h_stripe7_rev	-0.260	-0.050	-0.310	-0.063	0.211	-0.049	0.198	1	0
odd_-3	0.147	0.239	0.197	0.323	0.092	0.051	0.176	0	1
run_stripe5	0.487	0.589	0.604	0.646	0.102	0.117	0.159	0	0
oc_+7	0.449	0.545	0.614	0.603	0.096	0.165	0.154	1	0
odd_-5	-0.276	-0.184	-0.208	-0.173	0.092	0.068	0.104	1	0
gt_-6	0.401	0.416	0.524	0.500	0.015	0.122	0.098	0	0
run_-17	0.423	0.533	0.528	0.517	0.111	0.106	0.095	0	0
eve_stripe4_6	0.513	0.606	0.631	0.601	0.094	0.118	0.088	0	0
h_stripe34_rev	-0.118	0.005	-0.023	-0.030	0.123	0.095	0.088	0	0
oc_otd_early	0.496	0.548	0.561	0.579	0.052	0.066	0.083	1	0
run_-9	0.121	0.193	0.240	0.197	0.072	0.119	0.076	0	0
kni_+1	0.546	0.628	0.627	0.622	0.082	0.081	0.076	0	1
kni_-5	0.711	0.728	0.776	0.779	0.017	0.065	0.069	1	0
btd_head	0.571	0.518	0.627	0.638	-0.053	0.057	0.068	1	0
cnc_+5	0.528	0.554	0.628	0.585	0.026	0.100	0.058	1	0
nub_-2	0.735	0.816	0.829	0.784	0.082	0.094	0.049	0	1
gt_-10	0.631	0.642	0.640	0.668	0.011	0.008	0.036	1	0
kni_83_ru	0.601	0.741	0.639	0.637	0.139	0.038	0.035	0	1
fkh_-2	0.620	0.623	0.529	0.644	0.004	-0.090	0.024	0	0
hkb_ventral_elem	0.837	0.854	0.854	0.853	0.017	0.017	0.016	0	0
h_6_ru	0.635	0.610	0.593	0.648	-0.025	-0.042	0.013	0	0
run_stripe1	0.376	0.337	0.351	0.376	-0.039	-0.025	0.001	0	0
eve_1_ru	0.431	0.466	0.355	0.429	0.035	-0.076	-0.002	0	0
tll_K2	0.800	0.839	0.839	0.798	0.039	0.039	-0.002	0	0
prd_+4	0.549	0.464	0.526	0.546	-0.085	-0.023	-0.003	0	0
eve_stripe5	0.000	0.000	-0.041	-0.019	0.000	-0.041	-0.019	0	1
D_+4	0.693	0.649	0.668	0.656	-0.044	-0.025	-0.037	0	1
ftz+3	0.212	0.136	-0.001	0.157	-0.077	-0.214	-0.055	1	0
gt_-3	0.510	0.425	0.460	0.449	-0.085	-0.050	-0.061	0	0
pdm2_+1	-0.262	-0.353	-0.439	-0.338	-0.091	-0.177	-0.076	0	1
h_15_ru	0.605	0.517	0.513	0.521	-0.087	-0.091	-0.083	0	0
Kr_AD2_ru	-0.329	-0.551	-0.383	-0.417	-0.222	-0.054	-0.088	1	0
tll_P2	0.649	0.542	0.331	0.385	-0.107	-0.318	-0.264	0	0
eve_stripe2	0.404	0.115	0.140	0.132	-0.289	-0.263	-0.271	1	0
mean	0.298	0.393	0.359	0.428	0.095	0.061	0.130		
SE	0.008	0.008	0.009	0.007	0.005	0.005	0.005		

Table 1: **Perfomance of the dual models in comparison to the model using the "previous roles".** The **second** column shows the performance of the model, which uses the "previous" TF roles. The **third**, **forth** and **fifth** columns show the performance of the HbDual, KrDual and HbKrDual model, respectively. The **sixth**, **seventh** and **eighth** column shows the performance difference of the dual models to the reference model using "previous roles". Column **nine** and **ten** indicate for which CRM either Hb or Kr were used as activator, "1", or repressor "0". The **last two rows** show the mean and standard error over all 44 CRMs. All models are trained on the 44 CRMs *simultaneously*, out of five independent repeats the results of the best performing repeat is displayed.

Section 2 : Logo alignment for Br-Z4 and Bcd

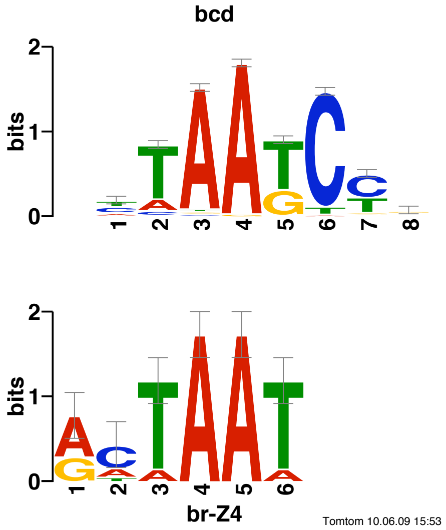

Figure 1: **Logo alignment between br-Z4 and bcd.** The figure shows the alignment between the PWMs of the two TFs as logo representation.

Section 3 : Transcriptional output of CRMs in Hb_act and Kr_act

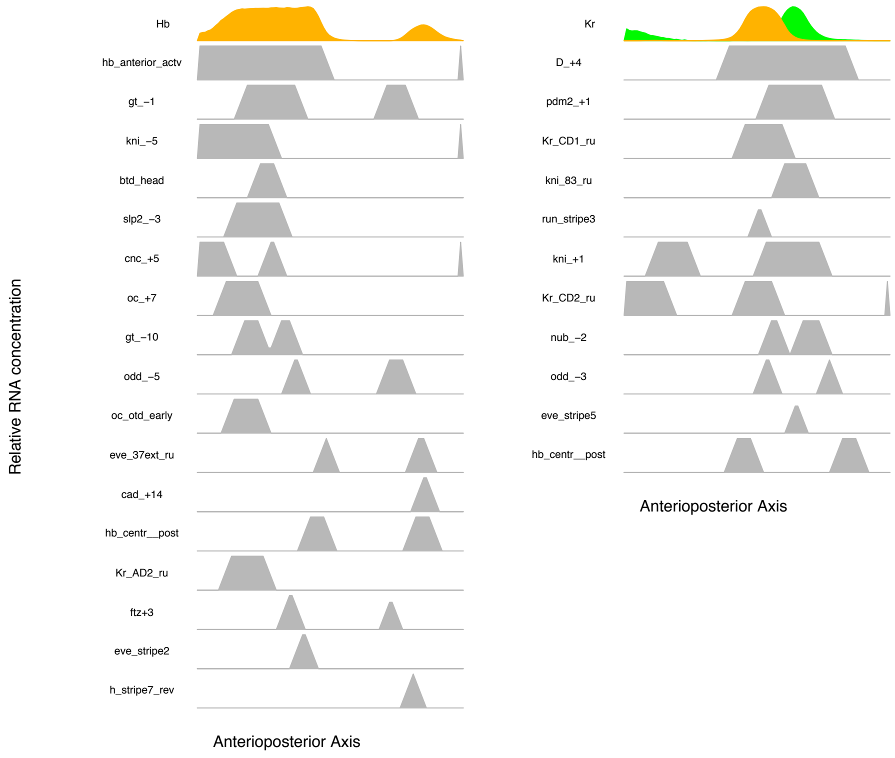

Figure 1: **Transcriptional output of the CRMs in Hb_act and Kr_act.** The figure shows the transcriptional output along the anterioposterior axis for the 17 CRMs in the Hb_act (left) and 11 in the Kr_act set (right). The protein concentration of Hb and Kr are shown in orange. The protein concentration of Kni is shown in green. The sets were determined according to the predictions of the SENSITIVITY method.

Section 4 : Predicted SUMOylation sites in *Drosophila* TFs

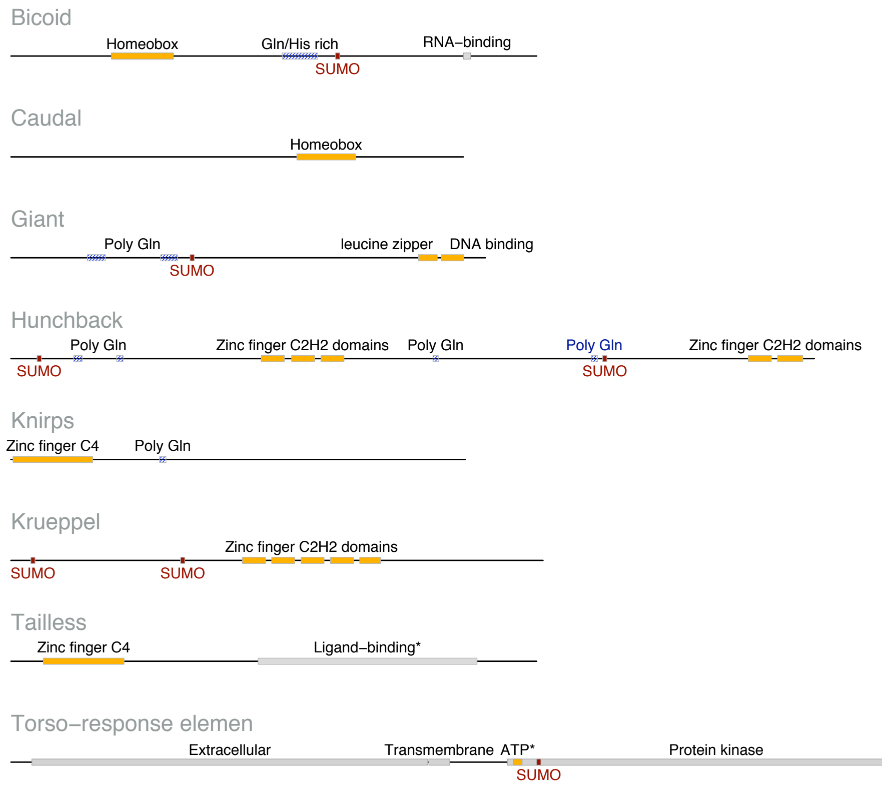

Figure 1: **SUMOylation sites in the protein sequence of the eight regulatory TFs.** The eight TFs are shown as horizontal lines. DNA-binding domains are shown in orange, other domains are shown in grey, glutamine (Gln) rich stretches are in blue and regular-expression hits for the SUMO consensus motif are shown in red. The domains and Gln-rich stretches are obtained from UniProt and predicted domains are taken from DBD Wilson et al. [2008] (highlighted with “*”).

Section 5 : Tested settings of the Reinitz model

model	*PWM type*	*t*	*bg*	*repres-sor range*	*simultaneously*		*individually*		*model similarity*	*over-fitting ratio*
					mean RMS (SE)	*mean CC (SE)*	*mean RMS (SE)*	*mean CC (SE)*		
Segal	Segal	-	uf	∞	0.21 (0.002)	0.59 (0.009)	-	-	-	-
Reinitz	Segal	9	uf	∞	0.32 (0.002)	0.22 (0.007)	0.23 (0.002)	0.60 (0.006)	0.799	2.73
				150	0.31 (0.002)	0.31 (0.008)	0.24 (0.002)	0.53 (0.007)	0.775	1.71
		6		∞	0.32 (0.002)	0.27 (0.006)	0.21 (0.002)	0.70 (0.005)	0.020	2.59
				150	0.31 (0.002)	0.29 (0.008)	0.24 (0.002)	0.62 (0.005)	0.002	2.14
	Shelf	9	dm	∞	0.32 (0.003)	0.13 (0.007)	0.23 (0.002)	0.51 (0.009)	0.141	3.92
				150	0.32 (0.003)	0.27 (0.008)	0.26 (0.003)	0.45 (0.009)	0.447	1.67
		6		∞	0.33 (0.003)	0.11 (0.006)	0.23 (0.003)	0.60 (0.008)	0.216	5.45
				150	0.44 (0.002)	0.12 (0.005)	0.44 (0.005)	0.36 (0.006)	0.505	3.00

Table 1: **Performance of the Reinitz model for different configuration settings.** The **second** column indicates whether Segal et al. [2008]'s tweaked PWMs or the conventional "off-the-shelf" PWMs were used. The **third** column specifies the PWM score threshold (in bits). Column **four** indicates which background model was used to calculate the log-odds scores - *dm* is the *D. melanogaster* specific background whereas *uf* is a uniform background. The **fifth** column shows whether short range (150 bp) or global (∞) repression was used. The **sixth** and **seventh** columns show the average CC for a model trained on all CRM *simultaneously*, whereas the **eighth** and **ninth** columns show the average CC over models trained on each CRM *individually*. Note, the objective during training is on the RMS error. A smaller RMS error is better, while for the CC a higher value is favorable. The *p*-value in column **ten** indicates how similar the performance between the Reinitz model and the Segal model is. Segal et al. [2008] grouped the predictions of their model in three classes "good", "fair", "poor". We record the CCs from the Reinitz model within those groups and employ the Kruskal-Wallis-Test to evaluate if the ranking of the three classes is reflected by the CCs. The resulting *p*-value states the confidence that both models have the same tendency to perform good or bad on certain CRMs. The **last** column states the over-fitting potential of the used settings calculated as the *simultaneous* CC/*individual* CC. In the main part of this study we use the settings (marked with "*") where the performance from training *individually* closest to the performance when trained *simultaneously*. As expected, the tweaked PWMs do not provide a large performance gain (0.31 vs 0.27) because they are adjusted to improve prediction accuracy when using the Segal model rather than providing a better general model for the TF binding profile.

Section 6 : Summary of all role determining methods

CRM	Bcd			Cad			Hb			Tll			Gt			Kr			Kni			TorRE			diffs
	∪	β	Δ	∪	β	Δ	∪	β	Δ	∪	β	Δ	∪	β	Δ	∪	β	Δ	∪	β	Δ	∪	β	Δ	
D_+4	−	−	+	+	+	+	−	−	−	−	−	NA	+	+	+	+	+	+	−	−	NA	−	−	NA	1
Kr_AD2_ru	−	−	NA	−	−	−	+	+	+	−	−	NA	−	+	+	+	−	NA	−	+	−	+	−	NA	4
Kr_CD1_ru	−	−	+	−	+	+	+	−	−	−	−	−	−	+	−	+	+	+	−	−	NA	+	−	NA	5
Kr_CD2_ru	+	+	+	−	+	−	−	−	−	−	−	NA	−	−	−	+	−	+	−	−	−	+	−	+	3
btd_head	+	+	+	−	+	NA	−	−	+	−	−	−	+	−	+	−	−	−	NA	−	NA	−	−	−	3
cad_+14	−	−	NA	+	+	+	+	−	+	+	+	+	+	+	−	−	−	NA	NA	−	NA	NA	+	NA	2
cnc_+5	+	+	+	+	+	NA	−	−	+	−	−	−	+	−	−	−	−	−	−	−	NA	+	−	+	3
eve_1_ru	+	+	+	+	+	+	−	−	NA	−	−	NA	NA	−	NA	−	−	−	−	−	NA	NA	−	NA	0
eve_37ext_ru	−	−	NA	+	+	+	+	−	+	+	−	NA	+	+	−	−	+	NA	−	−	−	−	−	−	4
eve_stripe2	+	+	+	+	+	−	−	−	+	−	−	−	−	−	−	−	−	−	−	−	−	−	−	NA	2
eve_stripe4_6	NA	−	NA	+	+	+	−	−	−	NA	+	NA	+	+	+	NA	−	NA	NA	−	NA	NA	+	NA	0
eve_stripe5	NA	−	NA	NA	+	NA	−	−	−	NA	−	NA	NA	+	NA	+	+	+	−	−	NA	NA	−	NA	0
fkh_-2	+	−	NA	+	+	+	−	−	−	−	+	NA	+	+	−	−	−	−	−	−	−	+	+	+	3
ftz+3	+	+	+	−	+	+	−	−	+	−	−	NA	+	−	NA	−	−	−	−	−	+	−	−	−	4
gt_-1	NA	−	NA	NA	+	NA	−	−	+	NA	+	NA	+	+	+	−	−	−	NA	−	NA	−	+	−	2
gt_-10	−	−	+	+	−	NA	+	+	+	+	−	NA	+	+	+	−	−	−	−	+	−	−	−	−	4
gt_-3	−	−	−	+	+	+	−	−	−	+	+	NA	+	+	+	−	−	−	−	−	+	−	+	NA	2
gt_-6	+	+	+	−	+	−	−	−	−	−	−	NA	−	−	−	−	−	−	−	−	−	+	−	+	2
h_15_ru	+	+	+	+	+	+	−	−	−	−	−	−	−	−	+	−	−	−	−	−	NA	−	−	NA	1
h_6_ru	−	−	−	+	+	+	−	−	−	+	+	+	NA	+	NA	−	−	−	−	−	NA	NA	+	NA	0
h_stripe34_rev	+	+	NA	+	+	+	−	−	−	−	−	NA	−	−	−	NA	−	NA	−	−	+	−	−	−	1
h_stripe7_rev	−	−	−	NA	−	NA	+	+	+	+	−	NA	NA	+	NA	−	−	−	+	+	+	NA	−	NA	1
hb_anterior_actv	+	+	+	−	+	NA	+	−	+	−	−	NA	−	−	+	−	−	−	+	−	NA	−	−	NA	4
hb_centr__post	−	+	NA	+	+	+	+	−	+	+	−	NA	+	−	−	−	−	+	−	−	−	−	−	NA	5
hkb_ventral_elem	NA	−	NA	NA	+	NA	NA	−	NA	NA	+	NA	NA	+	NA	NA	−	NA	NA	−	NA	+	+	+	0
kni_+1	+	+	+	−	+	+	−	−	−	−	−	NA	+	−	+	−	−	+	+	−	+	−	−	−	4
kni_-5	+	+	+	NA	+	NA	−	−	+	−	−	NA	NA	−	NA	−	−	−	NA	−	NA	−	−	+	2
kni_83_ru	−	+	+	+	+	+	−	−	−	−	−	−	+	−	NA	−	−	+	+	−	+	−	−	NA	4
knrl_+8	+	−	+	−	−	+	+	+	−	−	−	−	−	+	NA	−	−	NA	+	+	+	−	−	−	4
nub_-2	−	−	−	+	+	+	−	−	−	−	−	−	+	+	+	+	+	+	−	−	+	−	−	−	1
oc_+7	−	+	+	+	+	NA	+	−	+	+	−	NA	+	−	NA	−	−	−	+	−	NA	+	−	+	6
oc_otd_early	−	−	+	−	−	NA	+	+	+	+	−	NA	−	+	+	−	−	−	+	+	−	−	−	−	4
odd_-3	−	−	−	−	+	+	−	−	−	+	−	NA	+	+	NA	+	+	+	−	−	+	+	−	NA	4
odd_-5	−	−	−	+	−	NA	−	+	+	NA	−	NA	+	+	NA	−	−	−	NA	+	NA	−	−	−	2
pdm2_+1	−	−	−	+	+	NA	−	−	−	−	−	NA	+	+	+	+	+	+	−	−	NA	−	−	−	0
prd_+4	+	−	+	−	−	NA	−	+	NA	−	−	−	+	+	+	−	−	−	NA	+	NA	−	−	+	3
run_-17	NA	+	NA	+	+	+	−	−	−	NA	−	NA	−	−	−	NA	−	NA	−	−	NA	NA	−	NA	0
run_-9	NA	+	NA	+	+	+	−	−	−	NA	−	NA	−	−	−	NA	−	NA	−	−	−	NA	−	NA	0
run_stripe1	+	+	+	NA	+	NA	−	−	NA	−	−	−	NA	−	NA	−	−	−	−	−	−	NA	−	NA	0
run_stripe3	+	−	NA	+	+	+	−	−	−	−	−	NA	−	+	−	+	+	+	−	−	−	−	−	−	2
run_stripe5	NA	+	NA	+	+	+	−	−	−	−	−	−	NA	−	NA	−	−	−	NA	−	NA	−	−	−	0
slp2_-3	+	+	+	−	+	NA	−	−	+	−	−	NA	+	−	+	−	−	−	−	−	−	−	−	−	3
tll_K2	−	−	−	+	+	+	−	−	−	+	+	NA	−	+	−	−	−	−	−	−	−	+	+	+	1
tll_P2	−	−	−	−	+	−	−	−	−	+	+	NA	+	+	−	+	−	NA	−	−	−	+	+	+	3
activator	18	20	21	24	37	23	11	7	17	12	9	2	22	24	15	10	8	11	7	7	9	11	9	10	
repressor	19	24	9	14	7	5	32	37	23	25	35	11	14	20	15	29	36	23	28	37	15	24	35	15	
NA	7	0	14	6	0	16	1	0	4	7	0	31	8	0	14	5	0	10	9	0	20	9	0	19	
fold change	1.06	1.2	2.33	1.71	5.29	4.6	2.91	5.29	1.35	2.08	3.89	5.5	1.57	1.2	1.0	2.9	4.5	2.09	4.0	5.29	1.67	2.18	3.89	1.5	
role	s	s	s	s	+1	+1	s	-1	s	s	-1	-1	s	s	s	s	-1	s	-1	-1	s	s	-1	s	
diffs		11			14			16			8			20			7			12			11		

Table 1: **Roles for each of the 44 CRMs as determined by the different methods.** TF roles are repressor, "–", activator,"+", undetermined ,"NA". Methods are '∪" - SMALLEST-OPTIMAL, "β" - BEST-N and "Δ" - SENSITIVITY. Last row and column summarizes the disagreement between the three methods. "fold change" is the activator counts divided by the repressor counts (or vice versa) and "role" is the final role for the TF.

References

Eran Segal, Tali Raveh-Sadka, Mark Schroeder, Ulrich Unnerstall, and Ulrike Gaul. Predicting expression patterns from regulatory sequence includes Drosophila segmentation. *Nature*, 451(7178):535–540, Jan 2008. doi: 10.1038/nature06496. URL http://dx.doi.org/10.1038/nature06496.

Derek Wilson, Varodom Charoensawan, Sarah K Kummerfeld, and Sarah A Teichmann. DBD–taxonomically broad transcription factor predictions: new content and functionality. *Nucleic Acids Res*, 36(Database issue):D88–D92, Jan 2008. doi: 10.1093/nar/gkm964. URL http://dx.doi.org/10.1093/nar/gkm964.
